# EXPLORING ASSISTIVE TECHNOLOGY ACCESS AND USE AMONG OLDER ADULTS THROUGH SELF-DIRECTED HOME TOURS

**DOI:** 10.1093/geroni/igae098.2806

**Published:** 2024-12-31

**Authors:** Ben Mortenson, Chih-TIng (Gina) Tsai, Chin Wing Yu, Suzanne Huot, Laura Hurd

**Affiliations:** University of British Columbia, Vancouver, British Columbia, Canada; University of British Columbia, Vancouver, British Columbia, Canada; University of British Columbia, Vancouver, British Columbia, Canada; University of British Columbia, Vancouver, British Columbia, Canada; University of British Columbia, Vancouver, British Columbia, Canada

## Abstract

Assistive technology (AT) and home modifications play a crucial role in enhancing independence and quality of life for individuals with disabilities. Our aim was to explore access and use of AT for older adults through an innovative research methodology, virtual self-directed home tours. These interviews were held across three groups of participants (A) first-generation Chinese-speaking immigrants to Canada, (B) second and subsequent generations of Chinese-speaking immigrants, and (C) native-born English-speaking Canadian citizens of European descent. A total of 17 interviews were conducted (Group A=8, Group B=3, and Group C=6) through Zoom Video Communications. Participants were demographically diverse adults with disabilities living in Metro Vancouver, British Columbia (Age= 59 ± 15 years; 11 female and 6 male). Most participants had assistance from their caregivers to complete the interview. Three main themes were identified. (1) Enabling Participation described the transformative role of AT in optimizing participants’ ability to engage in activities that are meaningful to them independently. (2) User-Driven Do-It-Yourself Adaptations explored how participants developed cost effective alternatives by creatively adding their own modifications to existing AT. (3) Navigating Complexities and Hurdles encompassed the challenges with accessing and incorporating AT in their homes due to lack of adequate knowledge transfer from healthcare professionals, financial circumstances, and other systemic barriers that further delayed the implementation of home modifications. Chinese-speaking immigrants faced compound challenges due to language barriers. The insights gained from this study may inform strategies for improving the experiences of older adults accessing and using AT and home modifications.

